# A splicing variant of Merlin promotes metastasis in hepatocellular carcinoma

**DOI:** 10.1038/ncomms9457

**Published:** 2015-10-07

**Authors:** Zai-Li Luo, Shu-Qun Cheng, Jie Shi, Hui-Lu Zhang, Cun-Zhen Zhang, Hai-Yang Chen, Bi-Jun Qiu, Liang Tang, Cong-Li Hu, Hong-Yang Wang, Zhong Li

**Affiliations:** 1 International Cooperation Laboratory on Signal Transduction, Eastern Hepatobiiary Surgery Institute/Hospital, The Second Military Medical University, 225 Changhai Road, Shanghai 200433, China; 2 Institute of Biomedical Sciences, Fudan University, 138 Medical College Road, Shanghai 200032, China; 3 Department of Surgery, Eastern Hepatobiliary Surgery Institute/Hospital, The Second Military Medical University, 225 Changhai Road, Shanghai 200433, China; 4 Department of Oncology, Cancer Institute, The First Affiliated Hospital of Henan University of Science and Technology, 24 Jinghua Road, Luoyang 471003, China; 5 Renji Hospital, School of Medicine, Shanghai Jiao Tong University, 160 Pujian Road, Shanghai 200127, China; 6 Shanghai Center for Engineering Cell Therapy Research, 75 Qianyang Road, Shanghai 201805, China

## Abstract

Merlin, which is encoded by the tumour suppressor gene *Nf2*, plays a crucial role in tumorigenesis and metastasis. However, little is known about the functional importance of Merlin splicing forms. In this study, we show that Merlin is present at low levels in human hepatocellular carcinoma (HCC), particularly in metastatic tumours, where it is associated with a poor prognosis. Surprisingly, a splicing variant of Merlin that lacks exons 2, 3 and 4 (^Δ2–4^Merlin) is amplified in HCC and portal vein tumour thrombus (PVTT) specimens and in the CSQT2 cell line derived from PVTT. Our studies show that ^Δ2–4^Merlin interferes with the capacity of wild-type Merlin to bind β-catenin and ERM, and it is expressed in the cytoplasm rather than at the cell surface. Furthermore, ^Δ2–4^Merlin overexpression increases the expression levels of β-catenin and stemness-related genes, induces the epithelium–mesenchymal-transition phenotype promoting cell migration *in vitro* and the formation of lung metastasis *in vivo*. Our results indicate that the ^Δ2–4^Merlin variant disrupts the normal function of Merlin and promotes tumour metastasis.

## Abstract (web summary)

Merlin plays a crucial role as a tumour suppressor in liver tumorigenesis. Here, the authors show that a splicing variant of Merlin that lacks exons 2,3 and 4 (^Δ2–4^Merlin) is highly expressed in hepatocarcinoma and promotes tumour metastasis by interfering with the binding of wild-type Merlin to ß-catenin.

## Graphical abstract

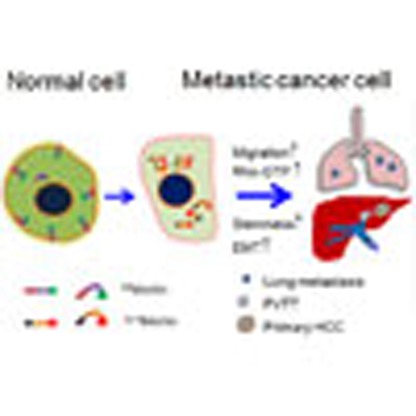

Hepatocellular carcinoma (HCC) is the fifth leading cause of death in patients with cancer. The development of portal vein tumour thrombus (PVTT) in HCC is rarely curable and associated with an extremely poor prognosis^1^. Approximately 50–80% of HCCs are accompanied by portal or hepatic vein invasion^2^. Cancer stem cells/tumour-initiating cells are associated with the formation of HCC, PVTT and tumour relapse due to their capacity for self-renewal and differentiation^3^.

Merlin (moesin-ezrin-radixin-like protein), encoded by the neurofibromatosis type 2 (*Nf2*) tumour suppressor gene, is a member of the band 4.1 family. As one of the most versatile tumour suppressors, it is capable of integrating several different mechanisms that regulate cell proliferation, motility, survival and signalling pathways^4^. Although mutations in *Nf2* gene are primarily associated with schwannomas and meningiomas^5^, it is not reportedly mutated in certain tumours such as those associated with breast, liver and colorectal cancer^6^,^7^. Wild-type Merlin (^wt^Merlin) is thought to be involved in cell-specific mechanisms of growth control^8^. In contrast to the narrow spectra of benign schwannoma and meningioma tumours in neurofibromatosis patients, *Nf2* heterozygous mice develop a variety of malignant tumours with high rates of metastasis^9^, which are dependent on the Merlin-mediated stabilization of adherens junctions^10^, the control of cadherin-mediated cell:cell contact^11^, and the activation of Rac^12^. The specific knockout of Merlin in mouse hepatocytes leads to both cholangiocellular and hepatocellular carcinoma^13^, which suggests that Merlin plays an important role in liver tumorigenesis. One of the mechanisms for the Merlin-mediated inhibition of tumour growth may be the control of organ size through the activation of YAP in mammals^14^,^15^. However, whether Merlin is involved in human HCC metastases remains largely unclear.

In this study, we first observe the expression of Merlin in HCC samples and find that the expression of ^wt^Merlin is relatively lower in tumour tissues than in adjacent non-tumour tissues. Next, we find that several spliced forms of Merlin are increased in HCC. Specifically, a splicing variant of Merlin lacking the sequences encoded by exons 2, 3, and 4 is identified and designated ^Δ2–4^Merlin. Compared with ^wt^Merlin, ^Δ2–4^Merlin losses its ability to interact with β-catenin and Ezrin/Radixin/Moesin (ERM) proteins through its N-terminal binding domain. The knockdown of Merlin or the overexpression of ^Δ2–4^Merlin promotes cell migration and invasion via an increase in Twist1. Furthermore, ^Δ2–4^Merlin also enhances the activation of β-catenin and stemness-related genes. Pulmonary metastatic mouse model shows that ^wt^Merlin reduces HCC cell-induced lung metastasis, while ^Δ2–4^Merlin promotes distant metastasis. Altogether, our results reveal that ^Δ2–4^Merlin functions as a tumour promoter. In particular, we discover that the new variant of Merlin promotes liver cancer metastasis by interfering with the tumour suppression role of ^wt^Merlin.

## Results

### Merlin expression in HCC is associated with patients′ survival

**Figure 1:**
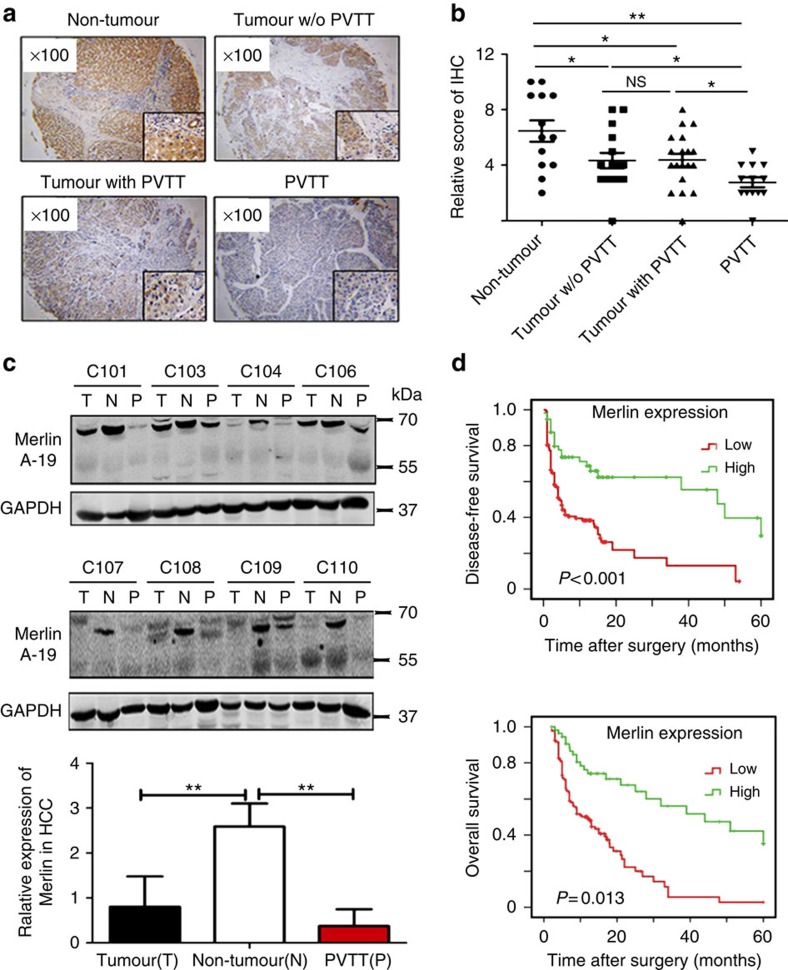
Low expression of Merlin in HCC and PVTT is associated with a cacoethic prognosis. (**a**) Merlin expression was decreased in HCC and PVTT in HCC tissue microarray sections. Merlin was stained in adjacent-tumour tissues, tumour tissues without PVTT, tumour tissues with PVTT and PVTT tissues (magnification: × 100; Small square magnification: × 400). (**b**) Merlin staining scores in the TMA (0–5 denote different degrees of IHC staining; 5 is the maximum and 0 is the minimum degree). The data are shown as the mean±s.d., 148 HCC with PVTT, 37 HCC without PVTT and 29 tumour-adjacent tissues specimens *P<0.05; **P<0.01, based on one-way ANOVA. (**c**) Western blots of Merlin proteins from eight pairs of HCC tumours with PVTT and tumour adjacent tissues using Merlin antibody. The quantification of the bands relative to GADPH is shown below the panels. (**d**) The disease-free and overall survival rates were obtained from a 5-year follow-up of 148 HCC patients based on their expression of Merlin. **P<0.01, based on the student's *t*-test.

To understand the role of Merlin in HCC, we detected the expression of Merlin with tissue-chips containing 148 samples of HCC with PVTT, 37 samples of HCC without PVTT, 29 samples of tumour-adjacent tissues and 16 samples of PVTT. We obtained and fixed 5-μm thick × φ3-mm sections on slides and stained them with an anti-Merlin antibody designed to target the N terminus of the Merlin protein. The expression of Merlin in these samples was quantified based on the percentage of positive cells and the density of staining by three people using 12 standard points, as previously reported. The overall results show that Merlin levels were expressed in the following order: tumour-adjacent tissues>tumours>PVTT (Fig. 1a,b). A western blot analysis of the tumour (T), non-tumour adjacent (N) and PVTT (P) tissues showed similar results when equivalent protein quantities were used (Fig. 1c). Subsequently, we analysed the relationships between Merlin and certain clinical and pathological characteristics. On the basis of the expression of Merlin in HCC and adjacent non-tumour tissues, the scores for Merlin in each sample were divided into high- and low-expression groups (mid index=5). Using analysis of variance (ANOVA), we found that the expression of Merlin was negatively associated with metastasis and the development of PVTT but was not significantly statistically related to sex, age, tumour size, HBV infection or tumor node metastasis (TNM) staging (Table 1). Kaplan–Meier estimates within 5 years of follow-up revealed that in 148 HCC patients a low expression of Merlin had shorter disease-free durations (*P*<0.001) and lower overall survival rates (*P*=0.013; Fig. 1d).

### Merlin limits cell migration and invasion

**Figure 2:**
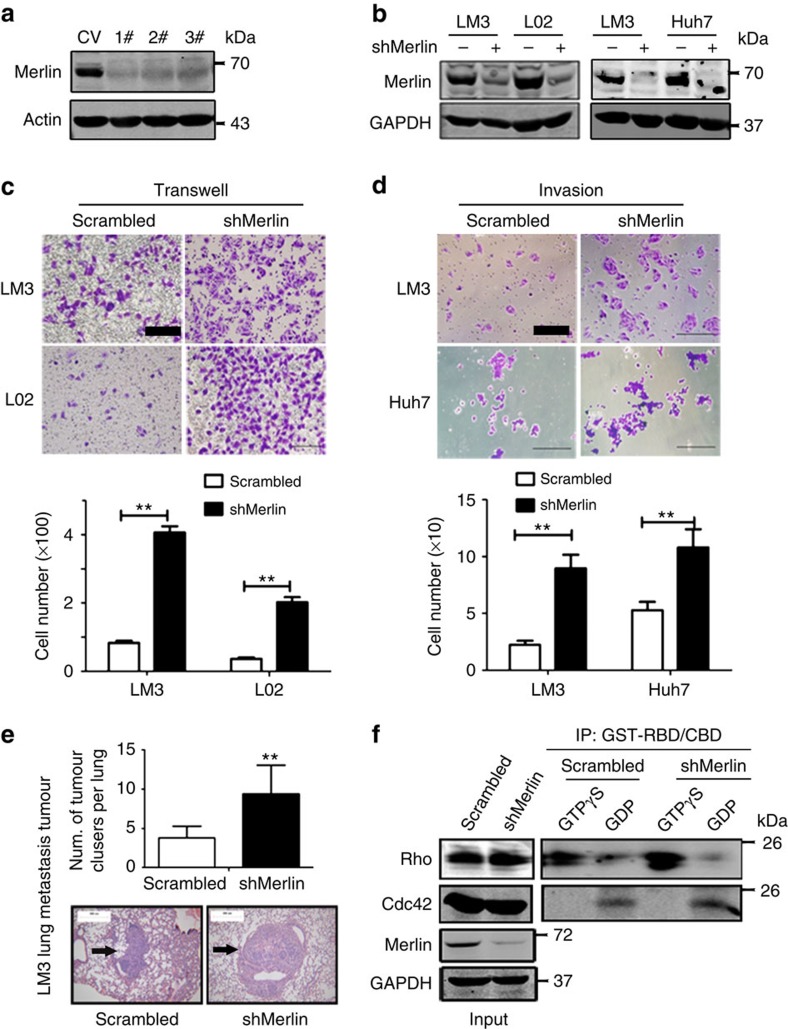
Interfering with the expression of Merlin promotes metastasis in HCC cell lines. (**a**) Lysates of HCCLM3 cells transfected with Merlin shRNAs were immunoblotted with the indicated antibodies. (**b**) Generation of shMerlin stable HCCLM3, Huh7 and L02 cell lines. (**c**) Transwell and (**d**) invasion assays were used to test the migration and invasion abilities of HCC cells. The respective infiltrated cell numbers are shown in the lower panes. (Scale bar, 50 μM). Results are shown as the mean±s.d., *n*=3. **P<0.01, ***P<0.001, based on the student's *t*-test. (**e**) Representative lung tissue sections of BALB/C mice killed at 13 weeks from each group are shown (H&E; Magnification : × 100, Scale bar, 300 μM ). The arrows indicate lung metastatic tumours (lower images). The average number of lung metastatic tumours per lung in each group (*n*=5) was calculated (upper panel). Results are shown as the mean±s.d., *n*=5. **P<0.01, based on the student's *t*-test. (**f**) Huh7 cells were treated *in vitro* with GTPγS or GDP to activate Rho and Cdc42. Cell lysates (50 μl at 1 mg ml^−1^) in Western blotting showed active Rho and Cdc42 levels in the shMerlin cell line and the control.

Tumour metastasis is known to be the major cause of death in cancer. Because the expression of Merlin was negatively associated with the development of PVTT and metastasis (Fig. 1, Table 1), we hypothesized that Merlin might be involved in tumour metastasis. Three shRNAs for Merlin were designed, two of which strongly interfered with the expression of Merlin (Fig. 2a). Thus, the #2 shRNA sequence was inserted into a lentivirus vector that was used to establish stable HCCLM3, Huh-7 and normal-like hepatocyte L02 cells expressing shMerlin (Fig. 2b). An analysis of cell migration showed that silencing Merlin in the L02 and HCCLM3 cells increased the number of migrated cells (Fig. 2c) and facilitated the invasion of HCCLM3 and Huh-7 cells (Fig. 2d). In a mouse pulmonary metastatic model, 1 × 10^6^ HCCLM3 cells expressing either shMerlin or scrambled RNA were injected into the caudal veins of three BALB/C nude male mice. The lungs of the mice were analysed for tumour nodules after 3 months. The HCCLM3 cells with downregulated Merlin exhibited increased numbers and volumes of lung metastatic tumours compared with control cells (Fig. 2e). These results suggest that Merlin plays a negative role in the regulation of cell motility and migration.

ERM is responsible for cytoskeleton reorganization and migration, which is regulated by the Rho family. Merlin decreases the activation of Rac1 (ref. 16). To detect whether knockdown of Merlin would regulate cell locomotion via the Rho family, we detected the activation of Rho and Cdc42 by using the specific binding domain GST-RBD for active Rho and GST-CBD for active Cdc42 in HCCLM3 cells. Although the knockdown of Merlin did not affect the expression of Rho and Cdc42 (Fig. 2f, left panels), it increased the activation of Rho but not Cdc42 (Fig. 2f, right panels). Therefore, downregulation of Merlin promotes migration possibly through Rho activation.

### Splicing variants of Merlin are amplified in HCC and PVTT

**Figure 3:**
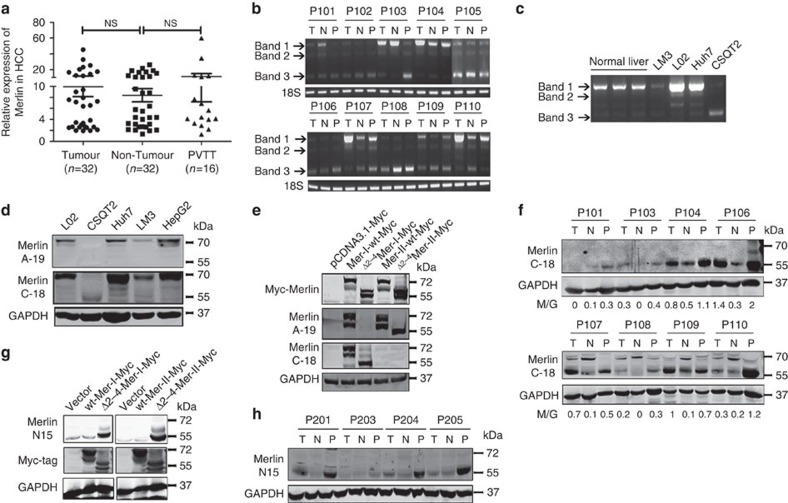
The alternative splicing of Merlin in HCC is determined by PCR and western blotting. (**a**) Expression of the *Nf2* gene in HCC, HCC without PVTT and HCC with PVTT tissues. (**b**) Detection of the sizes of *NF2* from HCC, adjacent and PVTT tissues by primers designed from the *Nf2* exon 1 and 5. (**c**) The splicing patterns of *Nf2* in normal liver, HCCLM3, Huh-7, CSQT2 and L02 cells. (**d**) Detection of expression of Merlin and its mutant in HCC cell lines (CSQT2, Huh-7, HCCLM3 and HepG2 cells), and hepatocyte L02 cells by antibodies (C-18 and C-19). (**e**) Detection of ^wt^Merlin and ^Δ2–4^Merlin by different antibodies. (**f**) Detection of the expression of Merlin and its mutant in HCC, PVTT and tumour adjacent tissues by antibody C-18. (**g**) Detection of ^Δ2–4^Merlin by specific antibody for ^Δ2–4^Merlin (Merlin-N15). (**h**) Detection of expression of ^Δ2–4^Merlin in HCC, PVTT and tumour adjacent tissues by Merlin N15 antibody. T: HCC tumour; N: non-tumour adjacent tissues; P: PVTT; M/G: Merlin/GAPDH density.

Observing the levels of *Nf2* mRNA in HCC, adjacent non-tumour and PVTT specimens by quantitative PCR (qPCR), we surprisingly found that there were no significant differences in expression (Fig. 3a). Considering that Merlin protein levels in HCC tissues were lower than those in adjacent non-tumour tissues, we speculated that the transcription of Merlin might be interrupted in HCC. On the basis of previous studiesshowing frequent splicingevents at the N terminus, a pair of primers was designed encompassing exons (1 to 5) of the *Nf2* open reading frame (sense: 5′- CAAGACGTTCACCGTGAGGAT -3′, antisense: 5′- GATTGCAAAGTAGTTCACACCG -3′). Reverse transcription PCR (RT–PCR) results revealed that, in addition to wild-type *Nf2*, there were several additional fragments present in normal liver tissues, HCC cell lines (HCCLM3, Huh-7) and PVTT-derived cells (CSQT2) (Fig. 3b,c), including at least three bands in the HCC tissues and cells (Fig. 3b,c). After sequencing these bands in Fig. 3c, we found that band 2 had a deleted exon 3, and band 3 lacked exons 2, 3 and 4 (Supplementary Fig. 1a,b). Interestingly, the Merlin variant lacking exons 2,3 and 4 appeared to be increased in CSQT2 cells (Fig. 3c). Western blots confirmed that this variant was sole band in CSQT2 cells (Fig. 3d), suggesting that it may be involved in the PVTT formation. So we selected it for further study and designated it ^Δ2–4^Merlin.

The difference between type I and type II Merlin is attributable to a difference in their C-terminus sequences wherein type I Merlin ends with exons 15 and 17, while type II Merlin ends with exons 16 and 17 (Supplementary Fig. 1b). To determine the functions of the Merlin variants in HCC, we generated constructs for full-length *Nf2* for type I and type II Merlin, as well as ^Δ2–4^Merlin from type I and type II Merlin. We found that the Merlin antibody A19 could recognize both type I and II ^wt^Merlin and type II ^Δ2–4^Merlin, whereas the Merlin antibody C18 could recognize only Type I ^wt^Merlin and Type I ^Δ2–4^Merlin (Fig. 3e). Subsequently, we found that both Type I and Type II ^wt^Merlin were expressed more highly in adjacent tumour tissues than in HCC tissues (Supplementary Fig. 1c). In the C19 antibody used samples in Fig. 1c, we detected with the C18 antibody and found that type I ^Δ2–4^Merlin was increased in HCC(T) and PVTT(P), and type I ^wt^Merlin was decreased in these HCC tissues (Fig. 3f). To determine the presence of ^Δ2–4^Merlin, we generated a polyclonal antibody specific to the combined regions of exons 1 and 5. This novel ^Δ2–4^Merlin antibody (Merlin N15) solely targeted ^Δ2–4^Merlin (Fig. 3g) and identified ^Δ2–4^Merlin expression in HCC and PVTT tissues (Fig. 3h). In comparison, as shown in Fig. 1c and Fig. 3f, the expression of type I ^Δ2–4^Merlin was higher than that of type II ^Δ2–4^Merlin in HCC. Because type II ^wt^Merlin does not function as a tumour suppressor^17^,^18^, we decided to explore the mechanisms of type I ^Δ2–4^Merlin and ^wt^Merlin in our study of liver tumourigenesis and metastasis.

### ^Δ2–4^Merlin promotes metastatic activity and EMT

**Figure 4:**
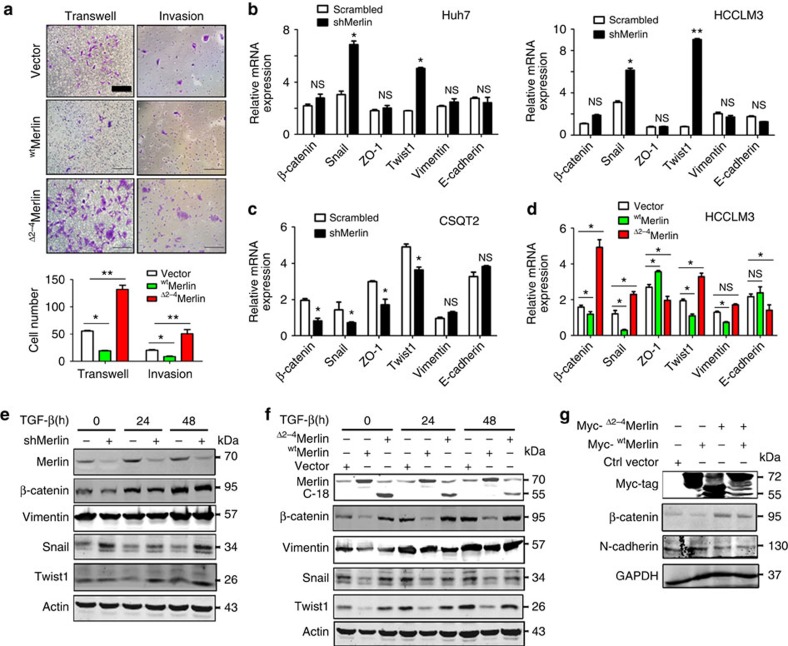
^Δ2–4^Merlin expression promotes cell migration and induces EMT. (**a**) ^Δ2–4^Merlin promotes HCCLM3 migration (Magnification: × 200; Scale bar, 50 μm). The number of migrated cells was counted (Mean±s.d., *n*=3) and is shown to the right of the panels. *P<0.05; **P<0.01, based on the student's *t*-test. (**b**) EMT-related genes were detected in Huh-7 and HCCLM3 cells with knockdown of Merlin. (**c**) EMT-related genes were detected in CSQT2 cells. (**d**) EMT-related genes were detected in ^wt^Mer (^wt^Merlin) or ^Δ2–4^Merlin expressing HCCLM3 cells. (**e**) Western analysis for EMT-related gene expression in HCCLM3 cells with knockdown of Merlin after treatment with TGF-β. (**f**) TGF-β induced expression of EMT-related genes in the background of the overexpression of ^wt^Merlin and ^Δ2–4^Merlin in HCCLM3 cells. (**g**) Western analysis showed β-catenin and N-cadherin expression in ^wt^Merlin, ^Δ2–4^Merlin or ^wt^Merlin/^Δ2–4^Merlin transiently transfected HCCLM3 cells with the indicated antibodies. *P<0.05; **P<0.01, based on the student's *t*-test.

To study the role of ^Δ2–4^Merlin in tumorigenesis, we generated expression vectors for ^wt^Merlin and ^Δ2–4^Merlin and expressed them in HCCLM3 cells. A migration assay showed that ^wt^Merlin inhibited cell migration and invasion, whereas ^Δ2–4^Merlin activated cell migration and invasion (Fig. 4a).

The epithelium–mesenchymal-transition (EMT) signifies that cancer cells are regaining their capacity to migrate and invade near and remote tissues. We found that Snail and Twist1 were increased in HCC Huh-7 (Fig. 4b left) and LM3 cells (Fig. 4b right) with shMerlin expression. Additional EMT-related genes, such as ZO-1, Vimentin, E-cadherin and β-catenin, were unaffected. In addition, the knockdown of Merlin in CSQT2 cells decreased the expression of β-catenin, Snail, ZO-1 and Twist1 (Fig. 4c). Furthermore, the expression of ^wt^Merlin in HCCLM3 cells decreased the expression of Snail, Twist1 and Vimentin, and slightly increased the expression of ZO-1 (Fig. 4d). In contrast, ^Δ2–4^Merlin reversed the effects of ^wt^Merlin (Fig. 4d). To confirm changes in these EMT-related genes with respect to protein levels, Western blotting was performed to analyse the expression of the EMT genes and β-catenin after stimulation with TGF-β, which is known to induce EMT. Silencing Merlin resulted in increases in Snail and Twist1 expression compared with scrambled RNA-expressing control cells (Fig. 4e). ^wt^Merlin suppressed Snail and Twist1 (Fig. 4f). Meanwhile, ^Δ2–4^Merlin blocked the endogenous Merlin-induced decrease in these protein levels (Fig. 4f). Vimentin seems not to be affected in the conditions of both overexpression and knockdown of Merlin (Fig. 4e,f). Taken together, these results suggest that Merlin is involved in tumour metastasis, and the loss of Merlin and/or increase of the ^Δ2–4^Merlin variant promotes tumour metastasis.

It is known that Merlin is involved in Wnt signalling through its interaction with β-catenin^10^,^19^. In stimulation with TGFβ, HCCLM3 cells showed similar patterns in, an increased expression of β-catenin (Fig. 4e). However, the stable overexpression of ^wt^Merlin reduced β-catenin expression, whereas the stable overexpression of ^Δ2–4^Merlin increased β-catenin expression (Fig. 4f). These results suggest that ^Δ2–4^Merlin may interfere ^wt^Merlin-mediated inhibition of β-catenin. In a rescue assay, when ^wt^Merlin, ^Δ2–4^Merlin and ^wt^Merlin/^Δ2–4^Merlin were transiently transfected into HCCLM3 cells, ^wt^Merlin reduced the expression of β-catenin and increased *N*-cadherin expression, whereas ^Δ2–4^Merlin evidently increased β-catenin expression and reduced *N*-cadherin expression (Fig. 4g). The transfection of ^Δ2–4^Merlin into cells expressing ^wt^Merlin blocked the ^wt^Merlin-induced reduction of β-catenin, although ^Δ2–4^Merlin expression appeared to be lower when it was jointly expressed with ^wt^Merlin compared with when it was expressed alone (Fig. 4g).

### Merlin regulates stemness feautures of cancer cells

**Figure 5:**
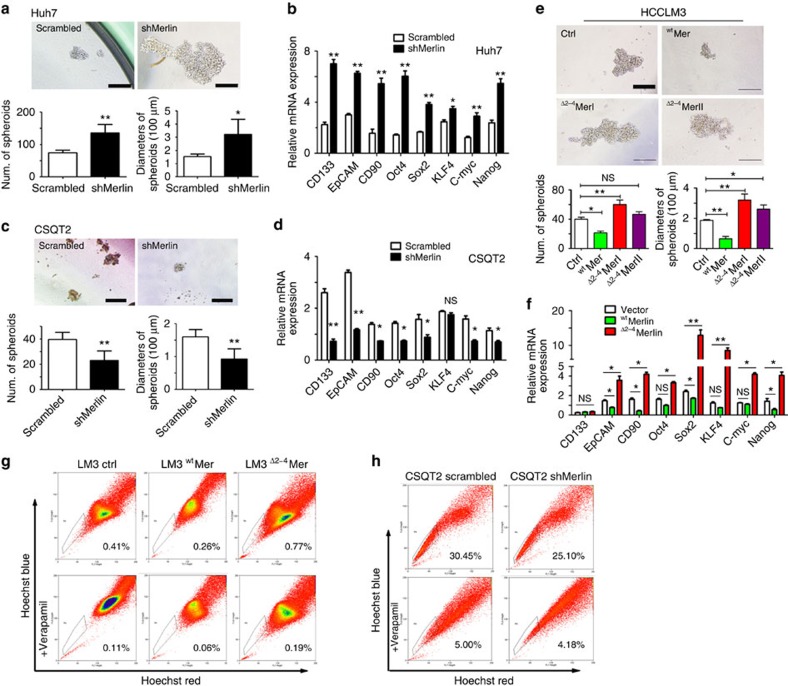
The silencing of Merlin or the expression of ^Δ2–4^Merlin promotes stemness activity. (**a**) shMerlin-expressing Huh-7 cells formed a greater number of and larger spheroids than scrambled-expressing Huh-7 cells in ultralow attachment plates without serum media. The quantities and sizes of the spheroids were measured and are shown under the each panel. Scal bar, 100 μm. (**b**) EMT-related genes were detected in Huh7-scrambled and Huh7-shMerlin cells. (**c**) The quantities and sizes of spheroids were measured in shMerlin-expressing and scrambled-expressing CSQT2. The knockdown of Merlin increased the number of spheroids in Huh-7 cells. Scal bar, 100 μm. (**d**) The expression of EMT-related genes was detected in CSQT2-scrambled and CSQT2-shMer cells. (**e**) Both ^Δ2–4^Mer type I and type II promoted the formation of spheroids of HCCLM3 cells compared with ^wt^Mer-expression cells. Scal bar, 100 μm. (**f**) Stemness genes were detected in ^wt^Mer-expressing and ^Δ2–4^Mer-expressing HCCLM3 cells. The above assays in **a**–**f** were repeated three times, respectively. The average values express as mean ±s.d. Statistic analysis in this figure was all based on the student's *t*-test. *P<005; **P<0.01; ***P<0.001. (**g**) The proportion of side population cells in empty vector, ^wt^Merlin, and ^Δ2–4^Merlin expressing HCCLM3 cells. (**h**) The proportion of side population cells in scrambled and shMerlin expressing CSQT2 cells.

Merlin has been shown to control progenitor homeostasis and tumorigenesis in the liver^13^. Accordingly, we observed spheroid formation in cells in which Merlin was knocked down. Huh-7 cells with shMerlin expression demonstrated increased spheroid numbers, and the number of spheroids with diameters greater than 100 μm was also increased in these cells (Fig. 5a), as well as the expression of stemness-related genes (Fig. 5b). As expected, the knockdown of Merlin in CSQT2 cells, which solely express ^Δ2–4^Merlin, decreased the number of spheroids and the expression of stemness genes compared with control cells (Fig. 5c,d). In contrast to the knockdown of Merlin, overexpression of either ^Δ2–4^Merlin type I (^Δ2–4^Mer I) or ^Δ2–4^Merlin type II (^Δ2–4^Mer II) in HCCLM3 cells resulted in an increase in spheroid formation (Fig. 5e) and the expression of stemness-related genes compared with control cells and ^wt^Merlin expressing cells (Fig. 5f). A side population (SP) analysis of the HCCLM3 cells (0.41%) showed that silencing Merlin reduced the positive rate of stem cells to 0.26%, whereas ^Δ2–4^Merlin increased the rate to 0.77%(Fig. 5g). At the same time, the knockdown of Merlin in CSQT2 cells decreased the SP rate (Fig. 5h). Thus, our data suggest that ^Δ2–4^Merlin can promote the property of cancer stemness.

### ^Δ2–4^Merlin no longer interacts with β-catenin and ERM

**Figure 6:**
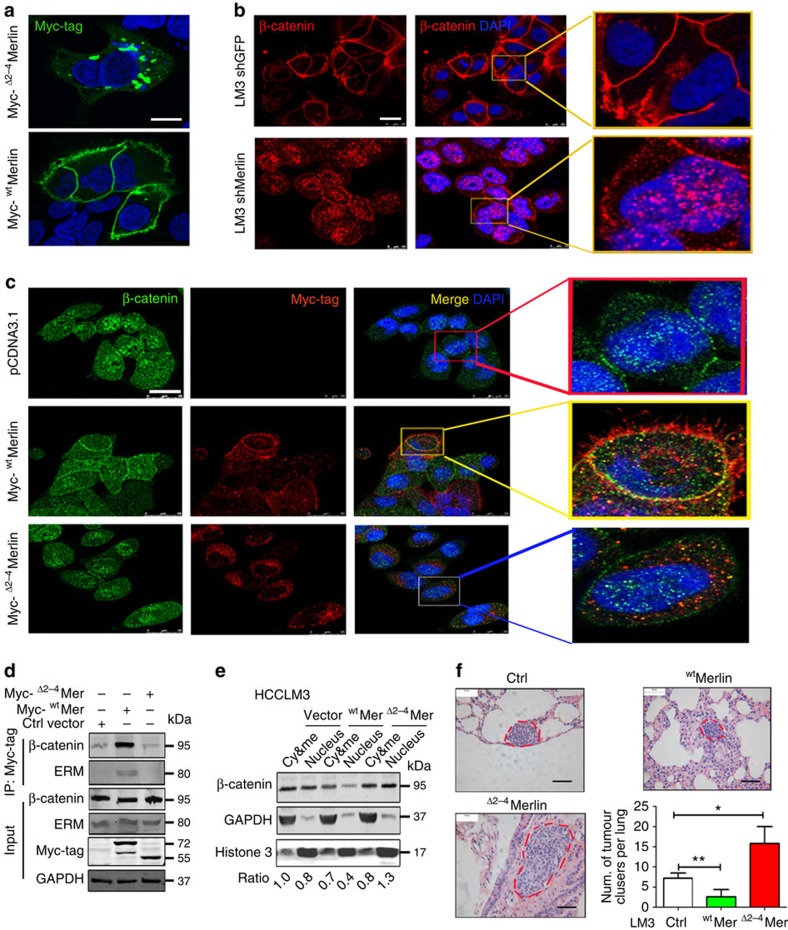
^Δ2–4^Merlin loses its functions of anchoring the membrane and binding to ERM and β-catenin. (**a**) Myc-tagged ^wt^Mer or Myc-tagged ^Δ2–4^Mer was expressed in HCCLM3 cells, and an anti-Myc antibody was used for immunofluorescence staining. (Scale bar, 20 μm). (**b**) Immunofluorescence analysis of β-catenin in HCCLM3 cells expressing shMerlin. DAPI was used for nuclear staining. Scale bar, 20 μm; Magnification for small square: × 400) (**c**) Confocal analysis for ^wt^Mer and ^Δ2-4^Mer in HCCLM3 cells. Myc-tagged ^wt^Mer or myc-tagged ^Δ2-4^Mer was expressed in HCCLM3 cells. The cells were stained with antibodies for β-catenin or Myc-tag. The nuclei were stained with DAPI. (Scale bar, 20 μm; Magnification for small square: × 400). (**d**) Immunoprecipitation assay for ^wt^Mer or ^Δ2–4^Mer with ERM and β-catenin. (**e**) The fractionation of ^wt^Mer and ^Δ2–4^Mer expressing HCCLM3 cells was performed and analysed by immunoblotting for β-catenin expression with the indicated antibodies. (**f**) ^Δ2-4^Mer- or ^wt^Mer-expressing HCCLM3 cells were injected into the caudal veins of BALB/C nude mice. The lung metastatic tumours were stained by H&E; Scale bar, 100 μm. The tumour numbers were counted and are shown to the right of the graphs (five mice per group). *P<0.05; **P<0.01, based on the student's *t*-test.

Merlin is associated with plasma membrane location, but ^Δ2–4^Merlin has lost its ability to anchor to the plasma membrane (Fig. 6a), suggesting that the domain encoded by exons 2–4 is responsible for membrane association. Merlin has been reported to bind to β-catenin and ERM. An immunoprecipitation assay revealed that ^wt^Merlin was associated with β-catenin on the plasma membrane, but the knockdown of Merlin caused the accumulation of β-catenin, primarily in the cytosol and nucleus (Fig. 6b). Immunoprecipitation analysis subsequently confirmed that ^wt^Merlin in HCCLM3 cells was bound to β-catenin and ERM but that ^Δ2–4^Merlin had lost this binding capacity (Fig. 6c). Furthermore, an immunofluorescence assay revealed that ^wt^Merlin was generally found to be bound with β-catenin on the plasma membrane, whereas ^Δ2–4^Merlin primarily accumulated in the cytosol surrounding the nuclei (Fig. 6d). ^Δ2–4^Merlin was increased in the cytosol possibly due to its dissociation with β-catenin and the plasma membrane. Thus, β-catenin was increased in the nuclei (Fig. 6d). A subcellular fractionation assay suggested that ^Δ2–4^Merlin promoted the translocation of β-catenin into the nucleus and that ^wt^Merlin limited this process (Fig. 6e). Taken together, these results suggest that the ^Δ2–4^Merlin variant disrupts the association of ^wt^Merlin with β-catenin or ERM, which in turn promotes the translocation of β-catenin into the nuclei.

To further study the role of ^Δ2–4^Merlin in tumour metastasis, we injected HCCLM3 cells expressing either ^wt^Merlin or ^Δ2–4^Merlin into the caudal veins of five BALB/C nude mice and observed their tumour nodules in the lungs. ^wt^Merlin overexpression reduced the size and number of lung nodules compared with control cells (Fig. 6f). However, the overexpression of ^Δ2–4^Merlin increased the formation of lung metastasis with respect to both the size and number of tumour nodules (Fig. 6f). Therefore, we conclude that ^Δ2–4^Merlin, an increased splicing variant of Nf2, plays a critical role in the liver tumorigenesis and metastasis.

## Discussion

*Nf2* heterozygous or homozygous mice have multiple malignant tumours. Merlin expression is markedly reduced in human malignant gliomas^20^. The loss of *Nf2* in the livers of mice leads to cholangiocellular and hepatocellular carcinoma^13^. However, less is known about the role of Merlin in the development and progression of human liver cancer. Our data not only track the levels of Merlin in HCC but also provide new evidence that an *Nf2* splicing variant in liver cancer is responsible for tumour proliferation and metastasis.

Merlin contains several functional domains. Its nuclear export sequence is encoded by exon 15, and a cytoplasmic retention domain is encoded by exon 2 (ref. 21). The deletion of exon 2 leads to the unrestricted entry of Merlin into the nucleus^21^,^22^. Less abundant variants such as ^Δ2^Merlin (missing exon 2), ^Δ3^Merlin (missing exon 3) and ^Δ2/3^Merlin (missing exons 2 and 3) demonstrate inactivity as tumour suppressors^23^,^24^. ^Δ2^Merlin, ^Δ3^Merlin, ^Δ2/3^Merlin, and even ^Δ15/16^Merlin exist in normal pleura^25^. The dominant effects of ^Δ2/3^Merlin are functionally associated with Schwann cell tumorigenesis in the presence of ^wt^Merlin^26^. ^Δ2–4^Merlin, which was first described in a malignant pleural mesothelioma cell line, is relatively prevalent (11.8%) and is principally expressed in malignant pleural mesothelioma^25^,^27^. However, the function of the splicing variant is unclear. We first studied the function of ^Δ2–4^Merlin in HCC and demonstrated that ^Δ2–4^Merlin is increased in HCC and PVTT. The accumulation of abnormal variants of Merlin in HCC should be taken into account when screening targets for cancer treatment. Given that the loss of Merlin occurs in highly malignant and metastatic tumours such as mesothelioma, colorectal cancer, melanoma and neurofibroma^24^,^25^,^28^,^29^,^30^, as well as HCC according to our data, we performed exome sequencing for *Nf2* gene and found no mutation present in human HCC, PVTT and non-tumour adjacent tissues (Supplementary Table 1). Therefore, it is believed that an increase in Merlin splicing variants is new characteristic associated with the liver metastasis and recurrence.

Aberrant splicing leading to the inactivation of Merlin drew considerable attention two decades ago^29^, and the loss of Merlin function can be achieved by truncating various locations in the protein^31^. However, most studies to date have focused on ^wt^Merlin or knockouts of *Nf2* in animal models; few reports have studied the functions of aberrant Merlin variants. Surprisingly, ^Δ2–4^Merlin was found to be expressed in HCC, specifically in the PVTT-derived cell line, which inspired us to explore the role of ^Δ2–4^Merlin in PVTT formation. Aberrant gene splicing can occur via exon skipping, intron retention and alternative 3′ or 5′ splice sites. The Merlin aberrant gene splicing in HCC is mainly attributable to exon skipping. Cancer-specific aberrant splicing of both oncogenes and tumour suppressor genes is associated with specific cancer types, and cancer shows a preference with respect to the selection of alternative splice-sites and the utilization of alternative splicing types^32^. Aberrant transcripts, often with truncated or missing domains, contribute to essential phenotypes that are associated with transformed cells^33^ and are a potential source of new diagnostic, prognostic, predictive and therapeutic tools for human cancer.

Merlin has a close relationship with migration and motility and serves as a linker between transmembrane proteins and the actin-cytoskeleton. An analysis of primary Schwannoma samples derived from neurofibromatosis patients showed that the activity of Pak1 was highly elevated^34^. The expression of a dominant-negative form of Merlin mimics *Nf2* deficiency and interferes with the ability of wild-type cells to assemble adherens junctions, confirming the specificity of Merlin's function in contact inhibition^10^. *Nf2*^−/−^ cells exhibit the characteristics of cells expressing activated Rac, which induces the phosphorylation of Merlin and decreases the association of Merlin with the cytoskeleton^35^. Merlin also inhibits mixed lineage kinase 3 (MLK3) activity by blocking the Cdc42–MLK3 interaction^36^. Merlin inhibits MLK3 activity by directly associating with MLK3 at its C-terminal residues from 340–590, preventing MLK3 from activating its downstream effectors B-Raf, extracellular signal-regulated kinase (ERK) and c-Jun N-terminal kinase (JNK), as well as its upstream activator, Cdc42 (ref. 37). In our study, the knockdown of Merlin increased RhoA activity, which is also responsible for cytoskeleton organization and polarization, but did not affect the expression and activation of Cdc42, which is similar to the results observed in HEK293 cells^37^.

Active Merlin maintains the beta-catenin and *N*-cadherin complex at the plasma membrane via direct regulation^19^. *Nf2* knockout mouse embryonic fibroblasts lose their contact inhibition of cell proliferation but exhibit significantly increased canonical Wnt signalling^38^. Dominant-negative TCF4 or Rac1 mutants, as well as small-molecule inhibition of Wnt, are able to curb *Nf2* deficiency-elicited confluent cell proliferation^38^. In this paper, we found that a variant form of Merlin can interfere with ^wt^Merlin-mediated functions and lead to an increase in β-catenin and promote its nuclear accumulation.

In summary, the expression of Merlin was found to be lower in HCC and PVTT versus non-tumour adjacent tissues, which is associated with remote metastasis and the formation of PVTT. In studying this mechanism, an increased number of splicing variants were found in HCC, in which ^Δ2–4^Merlin, lacking the sequences encoded by exons 2, 3 and 4, was identified and demonstrated to promote migration and metastasis. Compared with ^wt^Merlin, ^Δ2–4^Merlin has lost its ability to bind to β-catenin and ERM and promotes migration and invasion via an increase in Twist1. ^Δ2–4^Merlin also promotes stemness activity, potentially by increasing the expression of β-catenin and the nuclear accumulation and activities of other stemness genes (Sox2, OCT4, Klf4, C-myc and Nanog). Therefore, this Merlin alternative splicing variant (deletion of exons 2, 3 and 4) represents a novel mechanism for liver metastasis and PVTT formation.

## Methods

### Patients

Specimens including 185 primary HCC with or without PVTT from HCC patients who received curative surgery in the Eastern Hepatobiliary Surgery Hospital (Shanghai, China) from January 2000 to December 2003 were used for tissue-chips. 44 sets of HCC specimens (HCC, PVTT and non-tumour adjacent tissues) were used for qPCR or western blot analysis. A written informed consent was obtained from each patient and these studies were approved by the Ethics Boards of the Eastern Hepatobiliary Surgery Hospital.

### Cell cultures and stable cell line construction

The HCC cell lines HCCLM3, HepG2 and Huh7 as well as normal-type hepatocyte L02 cells were purchased from the Shanghai Cell Bank of the Chinese Academy of Sciences (Shanghai, China). The PVTT cell line CSQT2 was a gift from Prof. Shuqun Cheng, who established it in 2010 (ref. 39). All cells were routinely cultured in DMEM (Invitrogen) supplemented with 10% fetal bovine serum (FBS; Life Technologies) in an atmosphere of humidified air containing 5% CO_2_ at 37 °C. After seeding and incubation for 24 h, the cells were transfected with their respective plasmids using jet-PEI (Polyplus, New York, NY, USA) according to the manufacturer's protocol. Stable cell lines were constructed via the overexpression of plasmids or lentivirus vectors and selected with puromycin. The ^wt^Merlin and ^Δ2–4^Merlin expression plasmids were constructed by inserting fragments of the human type I Merlin and type II Merlin opening reading frames into the pCDNA^TM^3.1/*myc*-His(-)A-vector using EcorRI/BamHI. For the disruption of Merlin, lentivirus-GV248-shMerlin (5′- ACTTCAAAGATACTGACAT -3′) and control scrambled viruses were prepared by Genechem company (Shanghai, China).

### RT–PCR and real-time PCR analysis

Total RNA was extracted from tumour and non-tumour adjacent tissues and HCC cell lines with TRIzol (Invitrogen, Carlsbad, CA, USA). The reverse transcription of 2 μg of total RNA was performed using random primers (Roche Diagnostics, Penzberg, Germany) and SuperScript II reverse transcriptase (Invitrogen) according to the manufacturer's protocol. Because Merlin splicing frequently occurs at N terminus, two primers, sense: 5′- CAAGAGGAAGCAACCCAAG -3′ and antisense: 5′- GAAGCCAGGAGCACAGAAG -3′, were designed to lie outside the Merlin exon 1 and exon 5 reading frame for the amplification of Merlin transcripts in tumours and cells. Real-time PCR was performed to measure the expression of the transcripts of stemness or EMT-related genes with primers (Supplementary Table 2) in reaction solutions with iTaqSYBR Green Supermix (Bio-Rad Laboratories, Hercules, CA, USA).

### Immunoprecipitation and immunoblotting assays

Whole cell extracts and HCC tumour specimens were prepared in lysis buffer (20 mM Tris-HCl, pH 7.4, 150 mM NaCl , 10% glycerol, 0.2% Nonidet P-40, 1 mM EDTA, 1 mM ethylene glycol-bis[β-aminoethyl ether]- *N*,*N*,*N*′,*N*′-tetraacetic acid, 1 mM phenylmethylsulfonyl fluoride, 10 mM NaF, 5 mg ml^−1^ aprotinin, 20 mM leupeptin and 1 mM sodium orthovanadate) and centrifuged at 12,000 × *g* for 15 min. The supernatants were incubated with 2 μg of anti-Myc tagged or normal mouse immunoglobulin G (Santa Cruz Biotechnology Inc., Santa Cruz, CA, USA) for 8 h at 4 °C, followed by the addition of Protein A/G Plus-Agarose (Santa Cruz Biotechnology Inc.) for 2 h. Total and binding proteins were detected by western immunoblots. Immunoblots were performed using specific primary antibodies, following with a fluorescein-conjugated secondary antibody and then detected using an Odyssey fluorescence scanner (Li-Cor, Lincoln, NE, USA). The following primary antibodies were used: Merlin/NF2 (1:100, Santa Cruz Biotechnology, A19 sc331 rabbit and C18 sc332 rabbit), Twist1 (1:100, Santa Cruz Biotechnology, sc170453 mouse), β-actin (1:200, Santa Cruz Biotechnology, c-2 mouse), Myc-tag (1:1,000, CST, 71D10 mouse); β-catenin (1:1000, CST, D10A8 rabbit), Vimentin (1:1,000, Abcam, ab8978 mouse), ERM (1:1,000, Epitomics, 2054 rabbit) and Snail (1:1,000, Epitomics, L70G2 rabbit). Uncropped scans of immunoblots are provided in Supplementary Fig. 2.

### Immunofluorescence and immunohistochemistry

For immunofluorescence (IF) assay, cells were seeded into wells of glass-bottomed dishes and incubated for 24 h. Next, the cells were fixed with 4% paraformaldehyde (PFA) for 30 min after being rinsed twice with ice-cold PBS. The following primary antibodies were used: anti-Myc-tag (1:100, Cell Signaling Technology, 71D10 mouse) and β-catenin (1:100, CST, D10A8 rabbit). The secondary antibodies Alexa Fluor 488 (1:300, Invitrogen, Z25002 mouse or Z25302 rabbit) and Alexa Fluor 555 (1:300, Invitrogen Z25005 mouse or z25305 rabbit) were used. A Leica confocal microscope was used to capture the images.

For the immunohistochemistry (IHC) assays on tissue microarray (TMA), the collected paraffin embedded specimens including 148 HCC with PVTT, 37 HCC without PVTT and 29 tumour-adjacent tissues specimens were used to prepare the tissue-chips by Shanghai Outdo Biotech Co., Ltd. Tissue slides containing sections (5-μm thick × φ3-mm). Brifly, the tissue sections were dewaxed, rehydrated and then immersed in methanol containing 0.3% hydrogen peroxide (Sinopharm Chemical Reagent Co., Ltd., 10011218) for 30 min to block endogenous peroxidase activity. Subsequently, the sections were heated in a pressure cooker filled with 10 mM ethylenediaminetetraacetic (EDTA Sigma, 34550) buffer (pH 8.0) for 2 min. After cooling, the sections were incubated in 1% blocking serum (Chemicon, 20773) for 30 min to reduce nonspecific binding^40^. A primary antibody against Merlin/NF2 (1:100, Santa Cruz Biotechnology, sc331 rabbit) was used. The nuclei were labeled with DAPI. The secondary antibodies were HRP-conjugated goat anti-rabbit immunoglobulins (1:500, Santa Cruz, ).

### Fractionation of cells

To separate the cytosolic and nuclear proteins, the cells were collected in PBS and incubated for 20 min at 4 °C in S100/P100 buffer (20 mM Tris, 150 mM NaCl, 2.5 mM EDTA, 1 mM EGTA, 1 mM benzamidine, 4 μM leupeptin, 0.5 mM PMSF, 1 μM microcystin and 1 mM DTT at pH 7.5). After homogenization using a 26-gauge needle (Becton Dickinson), the nuclear pellets were collected by centrifugation for 2 min at 1,000 × *g* at 4 °C. The supernatants were collected as cytoplasmic and membrane fractions followed by centrifuging at 100,000 × *g* for 60 min at 4 °C. Equivalent nuclear, cytoplasmic and membrane fractions were then analysed via SDS–PAGE and immunoblotting.

### Spheroid assay

Hepatoma cells were plated at 4 × 10^3^ per ml in six-well ultralow attachment culture plates with DMEM/F12 (Invitrogen, #11320-033) containing B27 serum-free complement (Invitrogen, #17504-044), insulin (Sigma, solution, bovine, #I0516), bFGF (Invitrogen, #13256-029), and EGF (Sigma, #E4269) and were cultured for 10 days^41^. The quantities and sizes of the spheroids were measured in three independent experiments.

### Migration and invasion assays

Cell migration assay was performed with a transwell chamber (Corning Incorporated Costar, cat #3422,). Briefly, 1 × 10^5^ cells were seeded onto the upper surface of an 8.0-μm pore size transwell chamber for 24 h. The cell invasion assay was performed with an invasion chamber (BD Biosciences, cat #354480). The cells (1 × 10^6^) were loaded onto the upper chamber and the invasive cells which had invaded the lower surface of the membrane from the extracellular matrix layer after 48 h were fixed with methanol and stained with 0.1% crystal violet dye. Photographs of five randomly selected fields of the fixed cells were captured. The numbers of migrated cells were counted and expressed as mean±s.d. The experiments were repeated independently three times.

### Side population cells analysis using flow cytometry (FCM)

The cultured LM3 and CSQT2 cells at 70–80% confluence were digested with 0.25% Trypsin–EDTA and then suspended in 3% FBS/PBS. Volume of 1 × 10^6^ cells per ml were incubated with 20 μg ml^−1^ Hoechst33342 (Invitrogen, USA) either alone or with 25 μg ml^−1^ Verapamil (Sigma-Aldrich, USA) at 37 °C for 90 min. Cells were washed and suspended with ice cold 3% FBS/PBS containing 1 μg ml^−1^ propidium iodide (PI, Sigma-Aldrich, USA) as previously described^42^,^43^. FCM analysis was performed with a FACS apparatus MoFlo XDP (Beckman Coulter, USA).

### *In vivo* metastases assay

Scrambled RNA-, shMerlin-, ^wt^Mer-, ^Δ2–4^Mer- or control vector-expressing HCCLM3 cells (1 × 10^6^) were injected into the caudal veins of 5-week-old male BALB/C nude mice. Each group had five mice. All the mouse groups were killed after 2.5 months. The lungs of each mouse were separated and fixed for H&E staining. The average number of metastatic foci in each group was counted under a microscope. The protocols for the animal experiments were approved by the Ethics Boards of the Eastern Hepatobiliary Surgery Hospital.

### Statistical analysis

All the measurement data are expressed as the mean±s.d. The Pearson *χ*^2^-test was used to analyze the relationships between the expression of Merlin and the clinicopathologic features. A Kaplan–Meier analysis was used to assess the differences in survival rates. SPSS 16.0 software (SPSS Inc., Chicago, IL, USA) was also used for the above statistical analyses. A value of *P*<0.05 was considered to be significant.

## Additional information

**How to cite this article:** Luo, Z.-L. *et al*. A splicing variant of merlin promotes metastasis in hepatocellular carcinoma. *Nat. Commun.* 6:8457 doi: 10.1038/ncomms9457 (2015).

## Supplementary Material

Supplementary Information Supplementary Figures 1-2 and Supplementary Tables 1-2

## Figures and Tables

**Table 1:** Relationship of Merlin protein expression with clinical pathologic characteristics.

**Variable**	**No. of patients**	**Merlin expression in HCC**		***P*** **value**
		**Low**	**High**	
HCC tumour tissue				**<0.001**
Tumor without PVTT	37	12	25	
Tumour with PVTT	148	100	48	
HCC tumour tissue				**0.001**
Tumour	148	100	48	
Adjacent non-tumour	29	10	19	
Sex				0.169
Male	135	89	46	
Female	13	11	2	
Age (years)				0.205
>50	62	39	23	
≤50	86	61	25	
Tumour size (cm)				0.351
>5	120	79	41	
≤5	28	21	7	
HBsAg				0.335
Positive	129	89	40	
Negative	19	11	8	
TNM stage				0.427
I–II	102	66	36	
III–IV	42	31	11	
Distant metastasis				**0.001**
Yes	44	38	6	
No	104	62	42	

HCC, hepatocellular carcinoma. TNM, tumor node metastasis. Bold numbers indicate the statistical significance.

The Pearson *χ*^2^-test was used to analyze the relationships between the expression of Merlin and the clinicopathologic features in 148 HCC with PVTT, 37 HCC without PVTT and 29 tumour adjacent tissues. A value of *P*<0.05 was considered to be significant.
