# Supplementary material for: A splicing variant of Merlin promotes metastasis in hepatocellular carcinoma
Source: Nat Commun. 2015 Oct 7;6:8457. doi: 10.1038/ncomms9457 (PMC4633634; doi:10.1038/ncomms9457)
Supplement: Supplementary Information — Supplementary Figures 1-2 and Supplementary Tables 1-2 [file ncomms9457-s1.pdf]

Supplementary Figures

Supplementary Figure 1

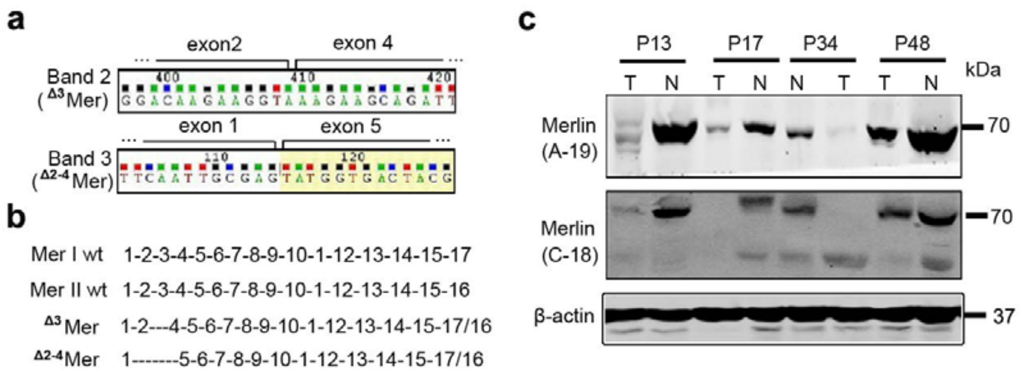

Supplementary Figure 1. Detection of $^{\Delta2\text{-}4}$Merlin in PVTT. (**a**) Sequence analysis of the fragments from Fig. 3b. Upper sequence showed deletion of exon 3. Lower sequence showed deletion of exon 2, 3 and 4. (**b**) Schematic overview of N-terminal frameshift of Merlin. $^{\Delta3}$Mer: $^{\Delta3}$Merlin; $^{\Delta2\text{-}4}$Mer: $^{\Delta2\text{-}4}$Merlin. (**c**) Western blots for Merlin proteins from 4 pairs of HCC tumor and tumor adjacent tissues (without PVTT) using antibody (Merlin A-19 and C-18).

Supplementary Figure 2

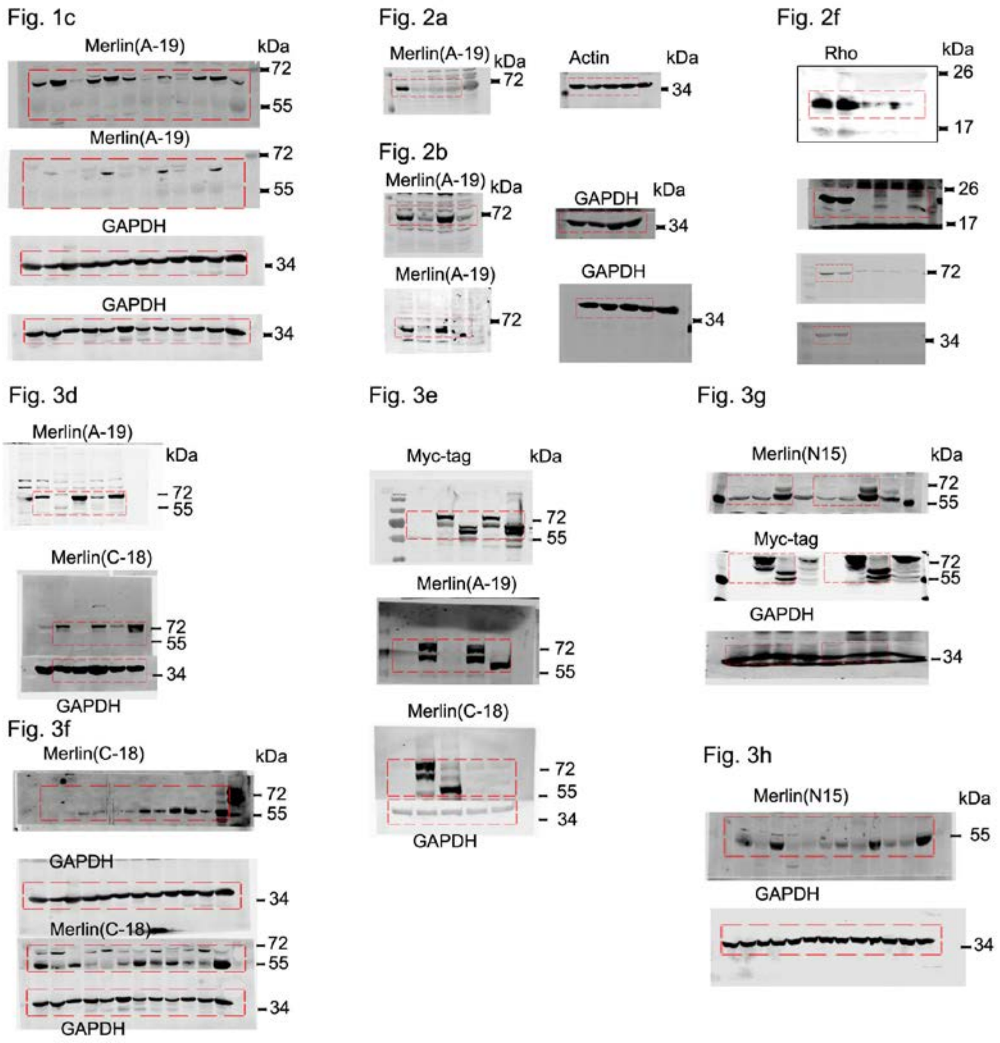

Supplementary Figure 2 (continued)

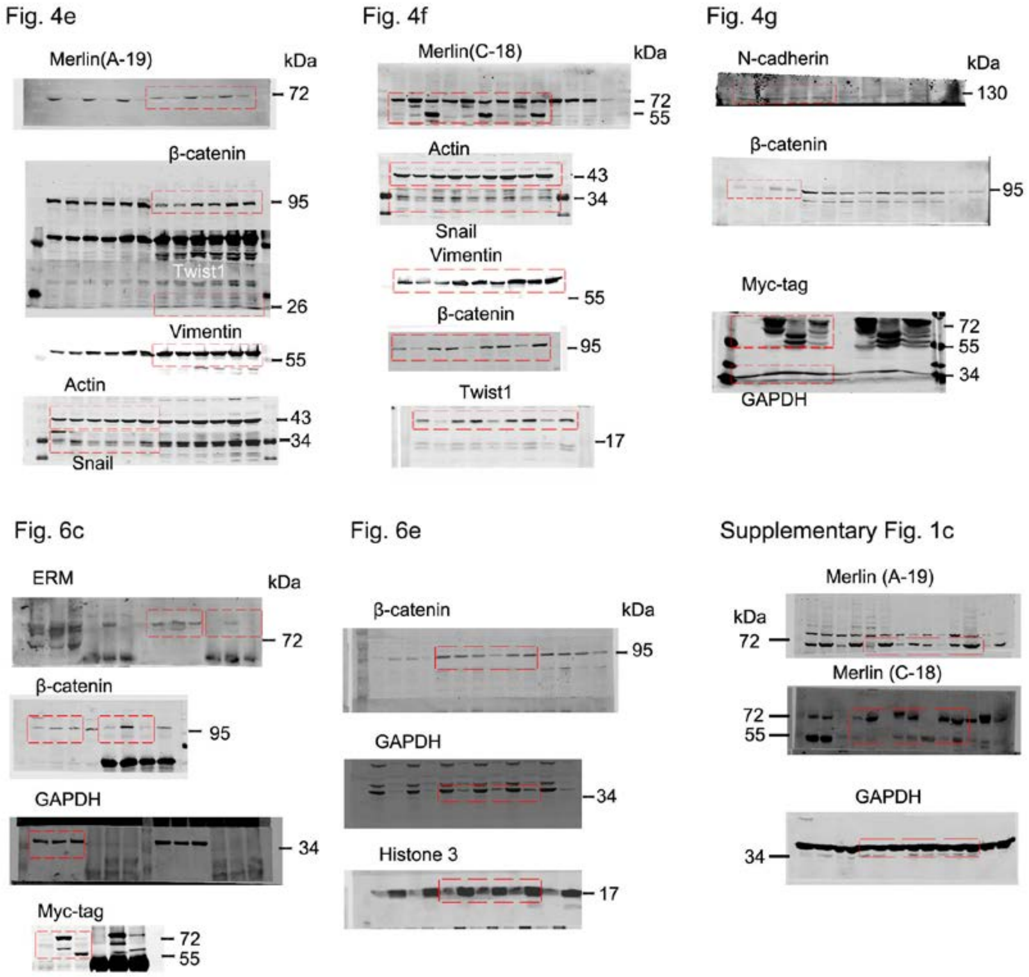

Supplementary Figure 2. The original immunoblots for Figure 1, 2, 3, 4, 6 and supplementary Figure 1. Red square represents where a panel in each immunoblot was cut. These images contain protein ladder markers that can partially be seen. We have also labeled the size of ladders on the right side according to the product instruction.

Supplementary Tables

Supplementary Table 1. Analysis of Nf2 exome in HCC patients.

Sample No. / Order	P2234 Tumor	P2234 Adjacent	P2234 PVTT	P1243 Tumor	P1243 Adjacent	P1243 PVTT	P3432 Tumor	P3432 Adjacent	P3432 PVTT
Exon 1	N	N	N	N	N	N	N	N	N
Exon2	N	N	N	N	N	N	N	N	N
Exon3	N	N	N	N	N	N	N	N	N
Exon4	N	N	N	N	N	N	N	N	N
Exon5	N	N	N	N	N	N	N	N	N
Exon6	N	N	N	N	N	N	N	N	N
Exon7	N	N	N	N	N	N	N	N	N
Exon8	N	N	N	N	N	N	N	N	N
Exon9	N	N	N	N	N	N	N	N	N
Exon10	N	N	N	N	N	N	N	N	N
Exon11	N	N	N	N	N	N	N	N	N
Exon12	N	N	N	N	N	N	N	N	N
Exon13	N	N	N	N	N	N	N	N	N
Exon14	N	N	N	N	N	N	N	N	N
Exon15	N	N	N	N	N	N	N	N	N
Exon16	N	N	N	N	N	N	N	N	N
Exon17	N	N	N	N	N	N	N	N	N

N: no mutation.

Supplementary Table 2. Sequences of PCR primers used for qPCR

Gene	Forward primer	Reverse primer
Oct4	AGTGAGAGGCAACCTGGAGA	ACACTCGGACCACATCCTTC
Merlin	CGAAGCACTGATGCGGTCTG	TGGGTTCATGGGCGGGTAC
CD133	CTGGGGCTGCTGTTTATTATTCTG	ACGCCTTGTCCTTGGTAGTGTTG
Sox2	CAAGATGCACAACTCGGAGA	GCTTAGCCTCGTCGATGAAC
KLF4	CCCACACAGGTGAGAAACCT	ATGTGTAAGGCGAGGTGGTC
Notch-1	TCCACCAGTTTGAATGGTCA	CGCAGAGGGTTGTATTGGTT
β-catenin	GAAACGGCTTTCAGTTGAGC	CTGGCCATATCCACCAGAGT
C-myc	ATGGCCCATTACAAAGCCG	TTTCTGGAGTAGCAGCTCCTAA
Nanong	TTTGTGGGCCTGAAGAAAACT	AGGGCTGTCCTGAATAAGCAG
BMI-1	CCACCTGATGTGTGTGCTTTG	TTCAGTAGTGGTCTGGTCTTGT
Vimentin	CTGGATTTCCTCTTCGTGGA	CGAAAACACCCTGCAATCTT
Snail	CATCTGAGTGGGTCTGGAGG	CTTCTCTAGGCCCTGGCTG
Fibronectin	AAACCAATTCTTGGAGCAGG	CCATAAAGGGCAACCAAGAG
CD90	CTAGTGGACCAGAGCCTTCG	GCACGTGCTTCTTTGTCTCA
EpCAM	CTGCCAAATGTTTGGTGATG	AAAGCCCATCATTGTTCTGG
18S	CGGCTACCACATCCAAGGAA	GCTGGAATTACCGCGGCT
